# Central Venous Catheter Based Closed Thoracic Drainage in the Treatment of Tuberculous Pleuritis

**DOI:** 10.12669/pjms.35.4.63

**Published:** 2019

**Authors:** Ling Song, Yueling Zhang, Qiong Jia

**Affiliations:** 1 Ling Song, Department of Cardiothoracic Surgery, Binzhou People’s Hospital, Binzhou, 256610, China; 2 Yueling Zhang, Department of Operating room, Binzhou People’s Hospital, Binzhou, 256610, China; 3 Qiong Jia, Department of Pharmacy, Binzhou Medical University Hospital, Binzhou, 256603, China

**Keywords:** Tuberculous pleuritis, Central venous catheter based closed thoracic drainage, Conventional thoracentesis and drainage

## Abstract

**Objective:**

To explore the clinical effect of central venous catheter closed thoracic drainage in the treatment of tuberculous pleurisy.

**Methods:**

One hundred and four patients with tuberculous pleurisy who were admitted to Binzhou People’s Hospital from August 2016 to August 2017 were divided into a control group and a treatment group according to random number table method, 52 each. The control group was treated with conventional pleural puncture and drainage, while the treatment group was treated with closed central venous catheter based thoracic drainage. The clinical efficacy, improvement time of clinical symptoms, total volume of drainage, pleural thickness, and improvement of quality of life and occurrence of adverse reactions were compared between the two groups.

**Results:**

Pleural effusion, fever and chest tightness of the treatment group disappeared earlier (P<0.05); the hospitalization time in the treatment group was less than that in the control group (P<0.05); the total amount of drainage in the treatment group was lower than that in the control group (P<0.05); the pleural thickness of the treatment group was higher than that in the control group (P<0.05); the quality of life score in the treatment group was significantly higher than that in the control group (P<0.05). The total effective rates of the treatment group and the control group were 93.5% and 85%, respectively, with a significant difference (P<0.05). The incidence of adverse reactions in the treatment group was significantly lower than that in the control group, with a significant difference (P<0.05).

**Conclusion:**

Central venous catheter based closed thoracic drainage is more effective than conventional thoracic puncture and drainage in the treatment of tuberculous pleurisy. It can accelerate the improvement of clinical symptoms, improve the quality of life of patients, and reduce the incidence of complications. It is worth popularizing and applying.

## INTRODUCTION

Tuberculous pleurisy is a pleural specific inflammatory reaction caused by the entry of tuberculosis bacillus and its metabolites into highly allergic pleural cavity. It is common extrapulmonary tuberculosis with a high clinical incidence, accounting for about 50% of the causes of pleural effusion.^1^ Patients with tuberculous pleurisy have high fibrin content in pleural effusion, which is easy to separate and form multilocular effusion deposited in pleura to aggravate pleural inflammation, inhibit the permeability of pleural vessels, induce pleural thickening, encapsulation and adhesion, and lead to dyspnea and pulmonary dysfunction.^2^,^3^

The treatment principle of tuberculous pleurisy is thoracic drainage, emptying inflammatory factors and fibrin, and giving necessary anti-tuberculosis drugs.^4^ The active pleural effusion emptying can reduce the toxic symptoms of tuberculosis virus, relieve the pressure of pleural effusion on the heart and lung lobes, thereby improving the function of the heart and lung.^5^ In the conventional thoracic puncture and drainage, 2-3 times of puncture per week was needed. This kind of repeated puncture is very harmful to patients, and due to the limitation of puncture means, drainage cannot be completely emptied, which may slow treatment progress and aggravate clinical symptoms.^6^,^7^ In recent years, a study has shown that central venous catheter drainage on the basis of standardized anti-tuberculosis treatment can reduce inflammation by eliminating pleural effusion quickly and early.^8^ Central venous catheter based closed thoracic drainage successfully overcomes the shortcomings of traditional drainage, such as time-consuming, laborious, small volume of drainage and large trauma. In central venous catheter based closed thoracic drainage, only one thoracic puncture is needed and the trauma is small; as a result, patients can move at will and show good tolerance. The speed of central venous catheter drainage can be adjusted, which can effectively reduce damages caused by suddenly declined intrathoracic pressure induced by chest fluid outflow. Moreover the drainage is continuous and the fluid flow can be easily controlled; hence the drainage is more complete.^9^,^10^

To further understand the clinical effect of central venous catheter based closed thoracic drainage in the treatment of tuberculous pleurisy, this study studied tuberculous pleurisy patients who underwent central venous catheter based closed thoracic drainage and analyzed the clinical efficacy.

## METHODS

One hundred and four patients with tuberculous pleurisy who were admitted to our hospital from August 2016 to August 2017 were selected as the study subjects. All patients had clinical symptoms such as hypodynamia, night sweating and chest pain during breathing and obvious pleural effusion characteristics, had no pulmonary parenchymal lesions under computed tomography (CT), and had 45 U/L higher adenosine deaminase (ADA) in pleural effusion. Inclusion criteria was symptoms such as low fever and night sweat in the afternoon, moderate or large amount of pleural effusion confirmed by CT examination and chest color Doppler ultrasonography, a large number of lymphocytes in the pleural effusion laboratory examination, tuberculosis foci in the lung, positive anti-organic nuclear antibody test result, positive tuberculin test result and tuberculosis characteristics changes in pleural biopsy or pleural biopsy. Exclusion criteria included dysfunction of organs such as the heart, lung and kidney, immunodeficiency disorders, mental disorders, other lung diseases, pregnant and lactating women and anti-tuberculosis drug allergy. According to the random number table method, the patients were divided into a control group and a treatment group. There were 27 males and 25 females in the treatment group; the age ranged from 19 to 67 years (average (43.73±15.29) years); the course of disease was 6 to 18 days ((12.82±4.33) days). In the control group, there were 28 males and 24 females; they aged from 20 to 67 years (average (43.89±16.42) years); the course of disease was 6 to 19 days (average of (12.95±4.63) days). There was no statistically significant difference between the two groups in sex, age and other baseline data (P>0.05). The study was approved by the ethics committee of our hospital, and all the selected patients signed the informed consent.

### Treatment Methods

Patients in the two groups were given conventional anti-tuberculosis treatment. Medicine was used according to the corresponding standards for drug use, and corresponding nursing operation was done during the perioperative period. The first part was reoperative nursing. Patients were informed with detailed introduction of the operation method, purpose, matters needing attention and potential complications of catheterization and drainage. They were asked to prepare well before catheterization to eliminate patients’ negative emotions such as anxiety and worry. The second part was intraoperative nursing. The sterile principle should be followed in the process of catheterization to avoid polluting sterile areas. During catheterization, the status of the patients was closely observed, and appropriate treatment was given if they felt uncomfortable. Catheters were fixed after catheterization. The third part was postoperative care. Drainage bottles were changed regularly, and the liquid in the bottles should not be too full. The drainage situation such as the color, quantity and nature of drainage fluid was evaluated. Pleural fluid bacterial culture was performed regularly to determine the time of extubation. Drainage tubes were checked at regular time to prevent drainage tubes falling off and being compressed. Puncture infection were prevented through corresponding measures such as avoiding showering and keep local skin clean and dry.

The patients in the control group were treated with conventional thoracic puncture and drainage. The patient took a sitting position, with the face towards the back of the chair and the forehead resting on the forearm. The patient was punctured according to the specifications of thoracic puncture. Before puncture, the puncture point was positioned by color Doppler ultrasonography. Puncture was performed according to the positioned point. The volume of drainage was no more than 600 mL at the first time and no more than 1000 mL at the second time. The interval of each time was 2-3 days. The drainage lasted until the pleural effusion was exhausted, i.e., color ultrasound showed that the volume of residual pleural effusion was lower than 1.5 cm.

### Closed thoracic drainage based o central venous catheter

The patient took sitting position. After skin disinfection, the skin of the puncture site was given local anesthesia, and then the site between the 5th and 6th rib at the midaxillary line was punctured. The puncture needle reached the thoracic cavity vertically. When the pleural effusion was seen, the puncture needle was inserted for 5-8 mm, and the guide wire was fixed at the central venous catheter and connected with the drainage bag. When the initial drainage volume exceeded 0.7 L, tube gripping was needed, for two hour Tube gripping for more than four hours was not allowed. The volume of following drainage was controlled within 1 L to avoid other adverse reactions induced by excessively large drainage volume. The drainage tube was removed when the drainage volume decreased to 0.

### Observational Indicators

The improvement time of clinical symptoms, including the disappearance time of pleural effusion, fever and chest distress, were observed. The total amount of pleural effusion was recorded. Pleural hypertrophy was examined by CT and pleural thickness was recorded. Quality of life instruments for cancer patients-breast cancer (QLICP-BR) was used; the higher the score, the higher the quality of life. The somatic condition, psychological condition, clinical symptoms and adverse reaction and social activity were scored. The clinical efficacy of two groups of patients was evaluated according to the following criteria.^12^ The clinical efficacy was evaluated according to X-ray chest film and ultrasound. The disease was evaluated as cured if no pleural effusion flew out and the pleura had no thickening. The treatment was considered as significantly effective if no pleural effusion flew out and only a small area of the pleura thickened or had mild costophrenic angle atresia and as effective if there was no large-area pleural effusion and the pleura was enclosed by effusion or had more serious thickening. The treatment was evaluated as ineffective if pleural effusion increased several days after drainage withdrawal. Total effective rate could be calculated using the following formula: overall effective rate=(number of cured cases+number of significantly effective cases+number of effective cases)/total number of cases*100%, the occurrence of adverse reaction was compared between the two groups.

### Statistical Methods

The data of the two groups were processed and analyzed by SPSS 21.0. The measurement data were expressed by Mean±SD. The comparison between the two groups was performed by t test. The counting data were expressed by rate (%) and X^2^ test. The difference was considered statistically significant if the value of P<0.05.

## RESULTS

The pleural effusion, fever and chest distress in the treatment group disappeared much earlier than those in the control group, and the difference was statistically significant (P<0.05, Table-I).

**Table I:** Disappearance time of clinical symptoms between the two groups.

Group	Treatment group	Control group	t	P
Disappearance time of pleural effusion	5.6±4.2	15.4±7.3	7. 747	<0.05
Disappearance time of fever	4.7±2.5	7.9±2.4	4. 664	<0.05
Disappearance time of chest distress	5.6±3.3	10.8±3.3	5.762	<0.05

The drainage volume of the treatment group was significantly lower than that of the control group, and the pleural thickness of the treatment group was higher than that of the control group; the differences were statistically significant (P<0.05, Table-II).

**Table II:** Drainage volume and pleural thickness between the two groups.

Group	Treatment group	Control group	t	P
Drainage volume (mL)	2900.1±385.2	3500.5±412.6	10.417	<0.05
Pleural thickness (mm)	1.1±0.2	1.3±0.4	2.973	<0.05

Scores of somatic condition, psychological function, clinical symptoms and adverse reactions and social functions in the treatment group were significantly higher than those of the control group, and there was a statistically significant difference (P<0.05, Table-III).

**Table III:** Scores of quality of life between the two groups.

Group	Treatment group	Control group	t	P
Somatic condition	25.4±3.6	19.9±2.4	4.169	<0.05
Psychological function	50.3±3.8	46.2±2.7	2.472	<0.05
Symptoms and adverse reactions	33.2±1.8	28.5±1.3	3.164	<0.05
Social function	42.2±3.3	38.4±3.1	3.551	<0.05

**Fig. 1:**
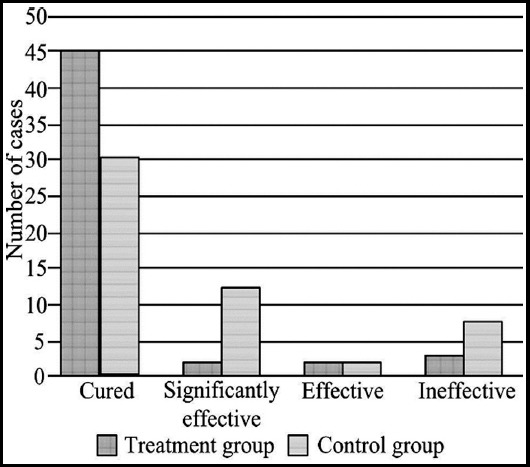
Clinical efficacy between the two groups.

The overall effective rate of the treatment group and control group was 84.2% (49/52) and 84.6% (44/52) respectively, and the difference was statistically significant (X^2^=6.186, P<0.05, Fig.1).

One patient in the treatment had encapsulated effusion; the incidence of adverse reactions in the treatment group was 1.9% (1/52). In the control group, three patients had encapsulated effusion, three patients had fever and two patients had subcutaneous emphysema; the incidence of adverse reactions in the control group was 15.4% (8/52). The incidence of adverse reactions of the treatment group was lower than that of the control group, and the difference was statistically significant (X^2^=4.915, P<0.05).

## DISCUSSION

In recent years, the incidence of tuberculosis has shown an increasing tendency, and the incidence of tuberculous pleurisy is also increasing. There are a large amount of a large number of lymphocytes, neutrophils, fibrin, monocytes and polykaryocytes in tuberculous pleural effusion. If pleural effusion is not timely drained, fibrin is easy to form fibrous strips to separate effusion, and the separated or alveolate effusion may be adhered to the pleura to induce pleural thickening; patients will have chest pain and difficult breath.^13^,^14^ If only anti-tuberculosis drug treatment is given to those patients, the slow absorption of pleural effusion and aggravated pleural thickening and adhesion may cause pulmonary insufficiency or lung failure.^15^ Therefore, on the basis of anti-tuberculosis treatment combined with repeated pleural puncture and drainage is usually adopted to treat tuberculous pleurisy in clinics, which has better efficacy than that of anti-tuberculosis drugs alone.^16^,^17^

In the past, pleural puncture was often used for drainage treatment in clinic, but the traditional thoracic puncture and drainage need multiple times of puncture. Repeated puncture may bring great pains to patients; hence patients’ compliance is poor, especially those who are elderly or weak, as they cannot stand puncture and drainage in a sitting position.^18^ Moreover the amount of drainage is limited in repeated puncture and drainage; therefore pleural effusion will disappear late, and moreover the incomplete drainage may increase the probability of pleural hypertrophy and pleural adhesion.^19^ Because of the low volume of the first drainage and the long interval between every time of drainage, the retention time of fibrin in the pleural cavity is increased, which increases the probability of encapsulated pleural effusion and pleural thickening?^20^ Risks of pneumothorax and hemothorax also increase if puncture and drainage are repeated.

Compared with the traditional thoracic puncture, central venous catheterization has many advantages in draining pleural effusion.^21^ It can relieve the pain caused by repeated puncture. It is easy to operate and maintain, reducing the nursing workload. Patients can take different positions, which is conducive to complete drainage of pleural effusion. After thoracic drainage, intrathoracic injection can still be carried out. The drainage volume and speed of pleural effusion can be adjusted to avoid the re-expansion pulmonary edema caused by excessive and rapid drainage. The results showed that the pleural effusion, fever and chest distress in the treatment group disappeared much earlier than those in the control group; scores of quality of life of the patients in the treatment group were significantly higher than those in the control group, indicating that the treatment group had more obvious relief of clinical symptoms and higher clinical effective rate; the incidence of adverse reactions in the treatment group was lower than that in the control group (P<0.05), which indicated that central venous catheterization based closed thoracic drainage was safe in the treatment of tuberculous pleurisy. It was also found that the total volume of drainage in the treatment group was significantly lower than that in the control group and the thickness of the pleura in the treatment group was higher than that in the control group, which was similar to the results of Wang.^22^ It indicated that central venous catheterization based closed thoracic drainage can improve the drainage of pleural effusion in a short time and play a very good role in hindering the formation of pleural thickening.

## CONCLUSION

Central venous catheterization based closed thoracic drainage on the basis of anti-tuberculosis treatment is effective for patients with pleural effusion caused by tuberculous pleurisy. Compared to the traditional thoracic puncture and drainage treatment, central venous catheterization based closed thoracic drainage can relieve symptoms more efficiently, significantly improve quality of life, and reduce incidence of complications. It is worth of popularization and application in clinics.

### Authors’ Contribution

**LS & YLZ:** Study design, data collection and analysis.

**YLZ & QJ:** Manuscript preparation, drafting and revising.

**LS:** Review and final approval of manuscript.
